# Supplementary material for: Targeting NKG2DL with Bispecific NKG2D–CD16 and NKG2D–CD3 Fusion Proteins on Triple–Negative Breast Cancer
Source: Int J Mol Sci. 2023 Aug 24;24(17):13156. doi: 10.3390/ijms241713156 (PMC10487695; doi:10.3390/ijms241713156)
Supplement: Supplementary file 1 [file ijms-24-13156-s001.zip › ijms-2556943-supplementary.pdf]

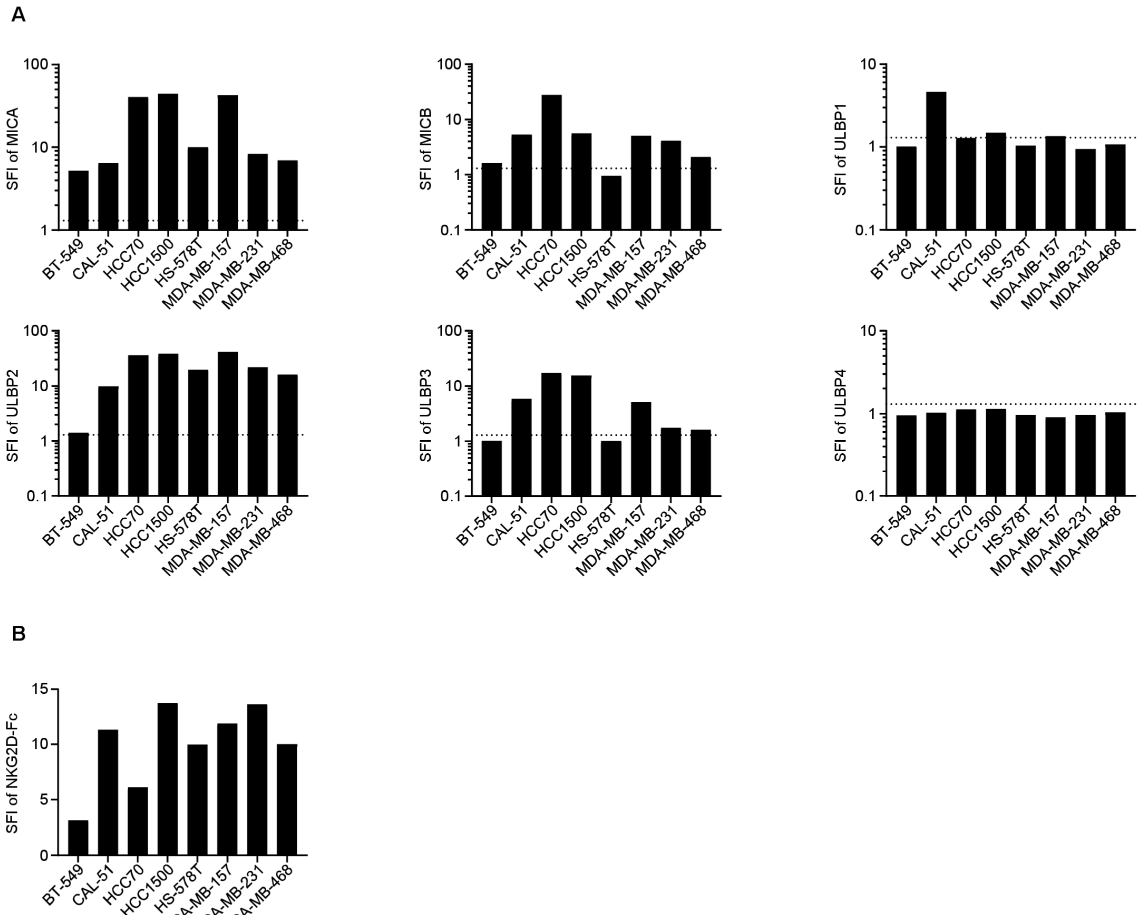

Supplemental Figure S1: Specific fluorescence intensity of NKG2DL

(A) TNBC cell lines were analyzed by flow cytometry for the specific fluorescence intensity (SFI) of the indicated NKG2DL. SFI levels above the dashed line show a SFI value >1.3 and are considered as NKG2DL positive. **(B)** The SFI of TNBC cell lines stained with the NKG2D–Fc was assessed by flow cytometry.

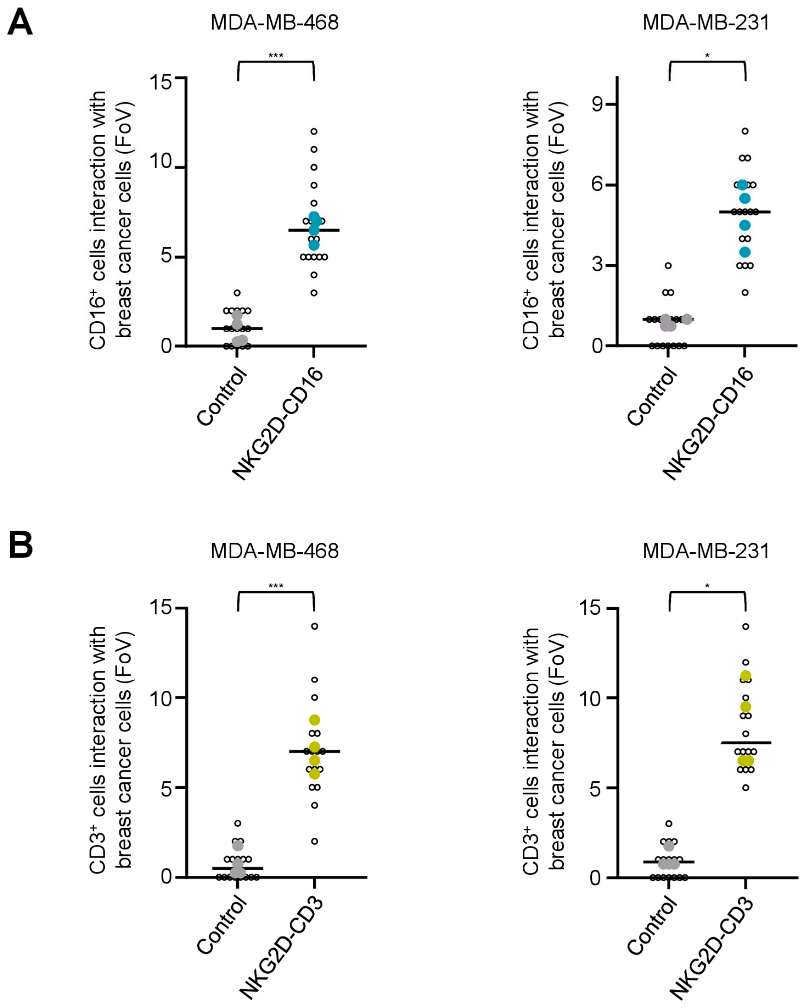

Supplemental Figure S2: Effector cell recruitment with the BFPs to TNBC cell lines

MDA−MB−468 cell line (left panel) and MDA−MB−231 cell line (right panel) were cultured with PBMC from healthy donors (n=4) at an E:T ratio of 5:1 in the presence of control or NKG2D−CD16 (blue) **(A)** or NKG2D−CD3 (green) **(B)** respectively. For each donor 4 field of views (FoV) were evaluated (small dots indicate FoV, large dots indicate the mean per donor).

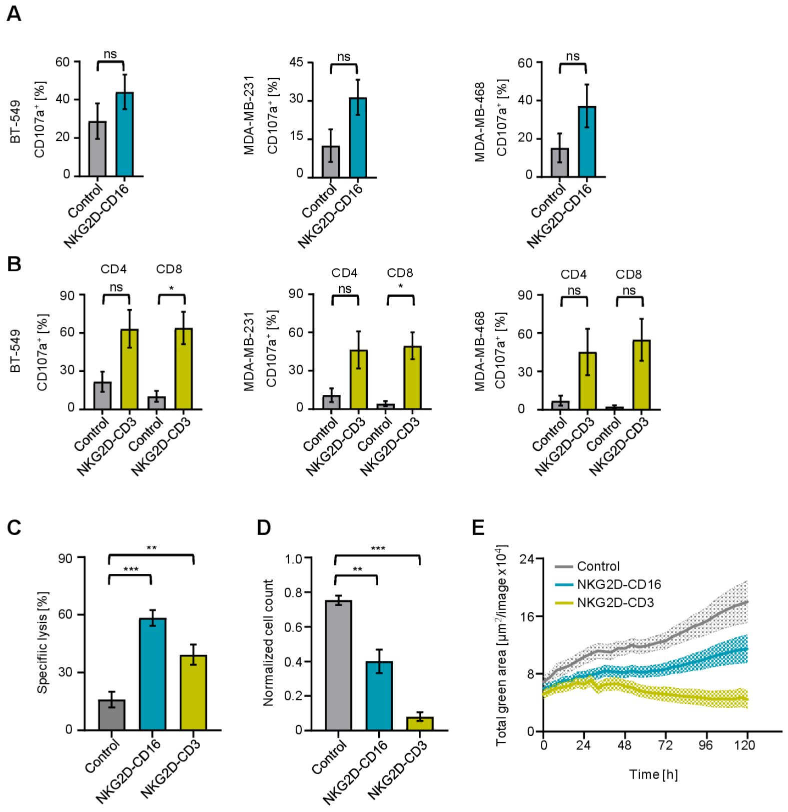

Supplemental Figure S3: Reactivity of T and NK cells from healthy donors against TNBC cell lines after NKG2D–CD16/CD3 treatment

TNBC tumor cells were incubated with PBMC from healthy donors (n=3) in the presence or absence of NKG2D–CD16/CD3 (2.5 µg/ml). **(A–B)** Degranulation of **(A)** NK cells and **(B)** T cells after coculture with indicated cell lines at an E:T ratio of 2.5:1 was determined by expression of CD107a after 4 h. **(C–E)** Cell lysis of 3 TNBC cell lines (BT–549, MDA–MB–231 and MDA–MB–468) by PBMC from healthy donors after the treatment with NKG2D–CD16 (blue) or NKG2D–CD3 (green) determined by **(C)** 2 h Europium assays with E:T ratio of 80:1, **(D)** flow cytometry–based lysis assay (E:T= 5:1) and **(E)** by a live cell imaging system with E:T= 5:1 is shown.

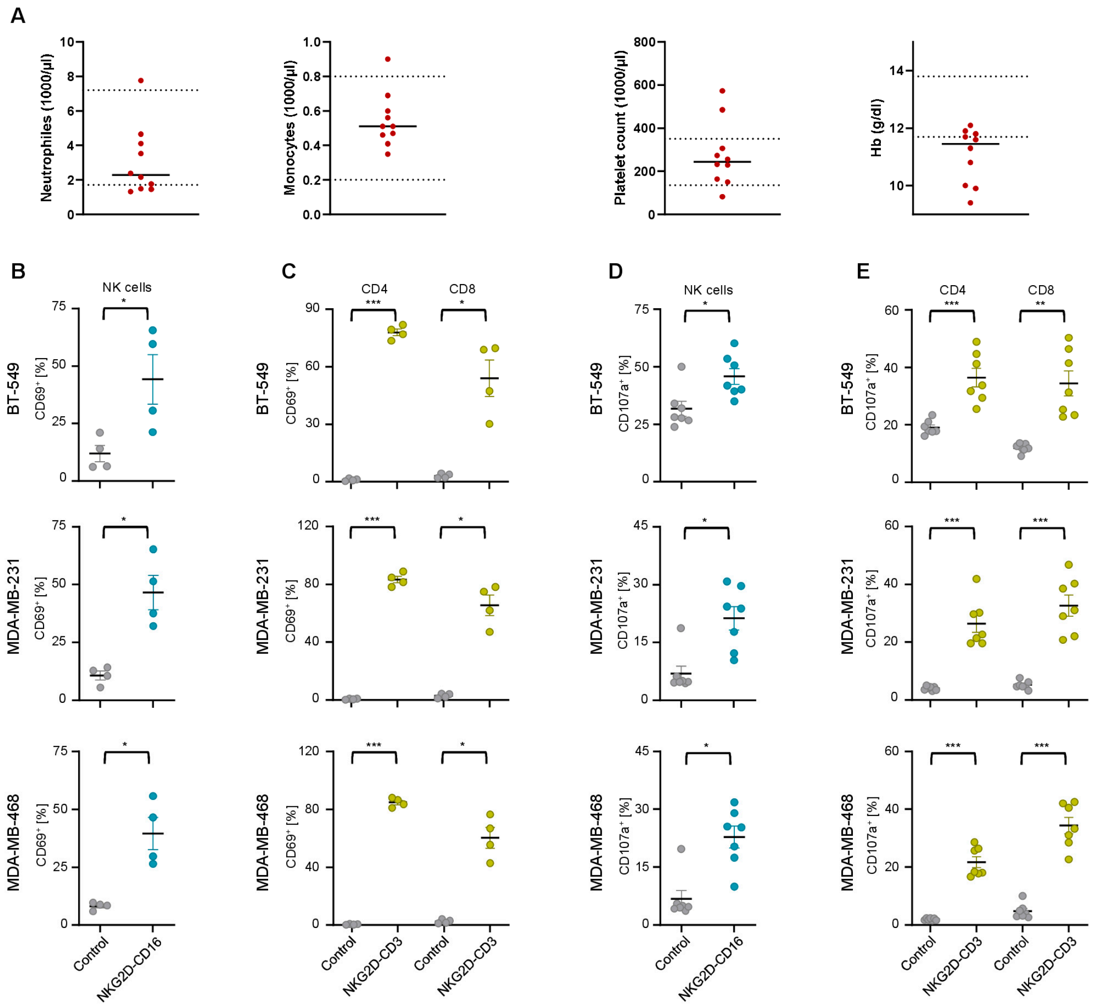

Supplemental Figure S4: Reactivity of T and NK cells of TNBC patients against TNBC cell lines

(A) Immune cell counts of neutrophils, monocytes, platelets and hemoglobin (Hb) concentration in TNBC samples at time point of PBMC collection. **(B–E)** Reactivity of NK and T cells against BT–549, MDA–MB–231 and MDA–MB–468 cells in response to NKG2D–CD16 (blue) and NKG2D–CD3 (green) treatment. PBMC of TNBC donors were cultured with TNBC cell lines at an E:T ratio of 2.5:1 in the presence or absence of NKG2D–CD16/CD3 (2.5 µg/ml). **(B, C)** Activation of NK cells **(B)** and $CD4^+$ and $CD8^+$T cells **(C)** of TNBC patients (n=4) was determined by expression level of CD69 after 24 h. **(D, E)** Degranulation of NK cells **(D)** and $CD4^+$ and $CD8^+$T cells **(E)** of TNBC patients (n=7) was determined by analysis of expression level of CD107a after 4 h.

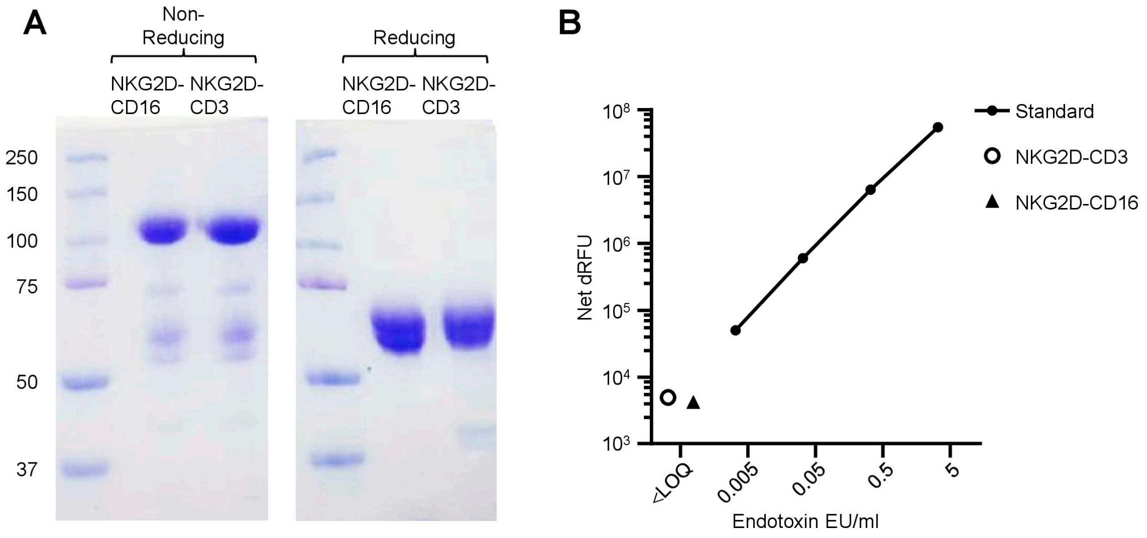

Supplemental Figure S5: Quality test for NKG2D–CD16/CD3 BFPs

(A) SDS page in non–reducing and reducing conditions and **(B)** endotoxin test results for both NKG2D–CD16/CD3 BFPs are shown.
